# High-Level *MYCN-*Amplified *RB1-*Proficient Retinoblastoma Tumors Retain Distinct Molecular Signatures

**DOI:** 10.1016/j.xops.2022.100188

**Published:** 2022-06-20

**Authors:** Khashayar Roohollahi, Yvonne de Jong, Saskia E. van Mil, Armida W.M. Fabius, Annette C. Moll, Josephine C. Dorsman

**Affiliations:** 1 Department of Human Genetics, Amsterdam UMC, Amsterdam, The Netherlands; 2 Department of Ophthalmology, Amsterdam UMC, Amsterdam, The Netherlands

**Keywords:** Methylation analysis, *MYCN*, RB1, Retinoblastoma, Transcriptomics, FC, fold change, GEO, Gene Expression Omnibus, LOH, loss of heterozygosity, RMA, robust multiarray average

## Abstract

**Purpose:**

Retinoblastomas are malignant eye tumors diagnosed in young children. Most retinoblastomas are genetically characterized by biallelic inactivation of the *RB1* gene. However, 1.5% of tumors demonstrate high-level amplification of the proto-oncogene *MYCN*. Patients with *MYCN*-amplified *RB1*-proficient retinoblastoma receive a diagnosis at an earlier age and show a clinically and histologically more malignant phenotype. This study aimed to identify genome-wide molecular features that distinguish this subtype from other retinoblastomas.

**Design:**

Cohort study.

**Participants:**

Forty-seven retinoblastoma tumors, comprising 36 *RB1*^–/–^, 4 *RB1*^+/–^, and 7 *RB1*^+/+^ tumors. In total, 5 retinoblastomas displayed high-level *MYCN* amplification, with 3 being *RB1*^+/+^, 1 being *RB1*^+/–^, and 1 being *RB1*^*–/–*^.

**Methods:**

Integrated analysis, based on gene expression, methylation, and methylation-expression correlations, was performed to identify distinct molecular components of *MYCN*-amplified *RB1*-proficient retinoblastomas compared with other retinoblastoma subtypes. The methylation and methylation-expression correlation analysis was initially conducted within a subset of samples (n = 15) for which methylation profiles were available. The significant findings were cross-validated in the entire cohort (n = 47) and in publicly available data.

**Main Outcome Measures:**

Differentially expressed genes/pathways, differentially methylated genes, and methylation-driven differential gene expression.

**Results:**

A large number of genes (n = 3155) were identified with distinct expression patterns in *MYCN*-amplified *RB1*-proficient retinoblastomas. The upregulated and downregulated genes were associated with translation and cell-cycle processes, respectively. Methylation analysis revealed distinct methylated patterns in *MYCN*-amplified *RB1*-proficient tumors, many of which showing significant impact on gene expression. Data integration identified a 40-gene expression signature with hypermethylated state resulting in a significant downregulation in *MYCN*-amplified *RB1*-proficient retinoblastomas. Cross-validation using the entire cohort and the public domain expression data verified the overall lower expression of these genes not only in retinoblastomas with a *MYCN*-amplified *RB1*-proficient background, but also in *MYCN-*amplified neuroblastomas. These include the metabolism-associated *TSTD1* gene and the cyclin-dependent kinase inhibitor gene *CDKN2C*.

**Conclusions:**

*MYCN*-amplified *RB1*-proficient retinoblastomas display significantly distinct molecular features compared with other retinoblastomas, including a set of 40 hypermethylation-driven downregulated genes. This gene set can give insight into the biology of *MYCN*-amplified retinoblastomas and may help us to understand the more aggressive clinical behavior.

Retinoblastoma is a malignant pediatric eye cancer with a median age at diagnosis of approximately 2 years.^1^ Incidence rates of retinoblastoma in Europe have been estimated at 1 in 13 844 live births.^2^ The diagnosis of retinoblastoma is by ophthalmoscopy and ultrasonography, because biopsies are contraindicated owing to the risk of metastasis.^3^^,^^4^ Retinoblastoma is highly curable when diagnosed in an early stage; however, current treatment options are not specifically targeting the molecular features of the tumor and are associated with local and systemic late effects.^4^^,^^5^ Retinoblastoma can develop as a heritable or sporadic tumor. Patients with heritable retinoblastoma (45%) demonstrate biallelic loss of the *RB1* gene, and in most patients, bilateral retinoblastoma develops. Nonhereditary retinoblastoma (55%) also develops because of biallelic inactivation of the *RB1* gene in approximately 97% of patients; however, a small percentage of patients with sporadic disease (approximately 1.5%) show *MYCN* amplification as the initiating event,^6^ whereas the disease of the remaining percentage (approximately 1.5%) remains unexplained. *MYCN*-amplified *RB1*-proficient (*MYCN*^A^ *RB1*^+/+^) retinoblastomas are diagnosed at an earlier age compared with classic *RB1*^*–/–*^ retinoblastomas and display more aggressive clinical behavior.^6^

Genetically, *MYCN*-amplified *RB1*^+/+^ retinoblastomas show fewer copy number changes compared with classic *RB1*^–/–^ retinoblastomas,^6^ and they are hypothesized to originate in an earlier retinal precursor cell^6^ compared with *RB1*^–/–^ retinoblastomas, which are thought to develop from a cone-photoreceptor precursor cell.^7^^,^^8^ In addition, their histologic appearance differs from the classic *RB1*^–/–^ retinoblastoma, showing an undifferentiated phenotype.^6^

Previously, we showed that *RB1*^–/–^ retinoblastomas are heterogeneous and express different levels of genes related to vision (photoreceptorness score), which is positively correlated with the amount of differentiation observed at the histologic level.^9^ Nondifferentiated tumors show a lower photoreceptorness score and upregulation of ribosome and mRNA synthesis genes. Interestingly, the two *MYCN*-amplified retinoblastomas that were included in this cohort clustered together with the undifferentiated *RB1*^–/–^ retinoblastomas.^9^

A recent study divided retinoblastomas into 2 different groups based on multiomics classification. Similarly to our previous study, the 2 groups showed a correlation between the level of differentiation and expression of vision-related genes, more specifically late cone markers. Again, *MYCN*-amplified retinoblastomas classified in the undifferentiated group.^10^ The present study dove deeper into the biology of the *MYCN* retinoblastoma subtype by correlating information of different molecular levels, defining *MYCN*-amplified *RB1*-proficient retinoblastoma as a unique retinoblastoma subtype with a distinct molecular background.

## Methods

### Patient Material and Characteristics

Retinoblastoma tumor material was previously collected.^9^^,^^11^ In brief, patient samples were collected after primary enucleation, before start of other treatments at the Amsterdam University Medical Center (UMC) and immediately frozen in liquid nitrogen and stored at –80° C. The study was conducted in accordance with the tenets of the Declaration of Helsinki and recommendations of the medical ethical committee of Amsterdam UMC, with a waiver of informed consent (identifiers, IRB00002991, FWA00017598; reference, 2014.360). Expression and copy number data were available for 47 patients, while methylation profiling was performed for a selection of 15 patients (Appendix 1)*.*

### RNA Extraction and Expression Profiling

RNA extraction and expression profiling were previously performed^9^ (GSE59983 [dataset ID of public data at GEO]). In brief, frozen tumor samples were homogenized in TRIzol with a rotor-stator homogenizer and RNA was extracted. DNase treatment was carried out and RNA was purified using the NucleoSpin RNA Clean-up XS kit (Macherey-Nagel). The Affymetrix HT HG U133 + PM96 microarray platform was used for expression profiling, which was performed at ServiceXS/Genomescan (Leiden, The Netherlands).

### Microarray Expression Analysis

The raw expression signal intensity CEL files were read into an R object by affy.^12^ A normalized expression matrix was created by the robust multiarray average (RMA) method embedded in the limma package.^13^ This was followed by generating a design matrix (i.e., *MYCN*^A^ *RB1*^PRO^; n = 4) versus the rest of the retinoblastoma cohort (n = 43), fitting the linear model and pairwise differential expression analysis by limma. To obtain gene-level differential expression, the probe-level-fold changes and *P* values were aggregated by the function of mean. Genes with the adjusted *P* value of < 0.05 were deemed significant. Hierarchical clustering was based on normalized log_2_ RMA expression values by the WARD2 method. Functional enrichment analysis was performed using the web-based functional annotation suite ToppGene (Cincinnati Childrens Hospital Medical Center).

### DNA Extraction and Sequencing

DNA extraction and sequencing were previously performed^11^ (EGAS00001001690 [ID number for published DNA sequence data]). In brief, genomic DNA was isolated using the NucleoSpin Tissue kit (Macherey-Nagel) or Wizard Genomic DNA Purification Kit (Promega). The TruSeq Nano DNA library prep kit (Illumina) was used for DNA end repair and ligation of sequencing and indexing adapters. Sequencing was performed using 125-bps paired-end sequencing (HiSeq, 2500; HT, v3/4; Illumina).

### DNA Copy Number Analysis

Copy number variation analysis was performed solely for the purpose of re-evaluating *MYCN* copy number levels and establishing the genomic copy number variable regions that need to be excluded before methylation analysis. The whole-exome sequence reads were used in somatic copy number analysis by CNVkit version 0.9.5.^14^ The pooled patient-matched diploid blood samples were used as reference in segmentation and calling copy number alternations in tumor samples. Genes with a log_2_ ratio (sample/control) of > 0.3 were considered as gain. Genes with a log_2_ ratio (sample/control) of < –0.3 were considered as loss. High-level amplification was defined as log_2_ ratio (sample/control) of > 2 (approximate absolute copy number, > 10).

### Methylation Profiling

Extracted genomic DNA (500 ng) was bisulfite-converted using the EZ DNA Methylation Gold Kit (Zymo Research). Converted samples were processed and hybridized on the Illumina Human Methylation450 BeadChip (Illumina) according to the manufacturer’s instructions. All steps were performed at ServiceXS/Genomescan. BeadChip images were scanned on the iScan system and data were extracted into GenomeStudio software version 2011.1 with Methylation Module version 1.9.0 using default settings. The number of detected genes per sample was between 484 945 and 485 435 of a maximum of 485 557 total genes per array.

### DNA Methylation Analysis

The raw methylation signal intensity files (IDAT file type) were read into a methylation intensity object using minifi.^15^ Data were normalized by quantile methods and probes with low quality (detection *P* values < 0.01) or high single nucleotide polymorphism frequency (> 0.01), as well as probes residing at X/Y chromosomes; frequently, copy number varied regions were filtered out. This was followed by calculating the methylated probe signal intensities as β and M values. β-Values were used in modeling data, hierarchical cluttering (WARD2), and correlation analysis. M values were used for the differential methylation testing between *MYCN*-amplified and *MYCN*-silent samples using limma. Gene-level methylation-fold changes and significance were obtained by aggregating the corresponding gene’s probe sets log_2_ fold change (FC) and *P* values by the function of the mean. Genes with the methylated log_2_ FC of < 0 and adjusted *P* value of < 0.05 were defined as hypomethylated. Genes with the methylated log_2_ FC of > 0 and adjusted *P* value of < 0.05 were defined as hypermethylated.

### DNA Methylation and mRNA Expression Correlation Analysis

Pearson correlation analysis between normalized methylation signal intensity (β-value) and normalized expression values (log_2_ RMA) were performed using the R/MvisAGE package.^16^ Correlations with *R* < 0 and a *q* value of < 0.05 were deemed significant. Because different methylation sites in relationship to CpG islands constitute different methylation amplitude and impact on expression, correlation analysis for shore, island, shelf, and open sea were performed separately. Subsequently, the end product of each analysis was compared and combined for data modeling. Functional annotation network analysis was performed by Cytoscape’s plug-in tool ClueGo.^17^^,^^18^

### Analysis of Public Domain Expression Data

The mRNA-expression microarray CEL files from an independent retinoblastoma cohort comprising 61 primary tumor samples were downloaded from Gene Expression Omnibus (GEO) (GSE58785 [dataset ID of public data at GEO]).^10^ The expression microarray CEL files from a neuroblastoma cohort comprising 88 tumors were downloaded from GEO (GSE16476 [dataset ID of public data at GEO]).^19^ The data normalization and the differential expression testing were conducted via the same tools and methodologies as used in the analysis of the in-house expression data.

### Data Availability

Expression data were previously published and available at GEO under database number GSE59983.^9^ Whole exome sequencing data also was previously published and available at EGAS00001001690 (ID number for published DNA sequence data).^11^ Publicly available data comprising mRNA expression data of 61 retinoblastomas was downloaded from the GEO under database number GSE58785.^10^ Methylation data are deposited in the GEO repository.

## Results

This study relied on a previously described retinoblastoma cohort comprising 47 tumor samples (Appendix 1).^9^ The *RB1* mutation and copy number status and *MYCN* somatic copy number levels were determined by whole exome sequencing. In total, 36 of 47 retinoblastomas indicated RB1^–/–^ status, 12 of which were caused by loss of heterozygosity of a nonsynonymous or high-impact germline variant. Four retinoblastoma tumors displayed *RB1*^–/+^ status and 7 exhibited *RB1*^+/+^ genotypes. Somatic copy number analysis identified 5 retinoblastoma tumors with high-level *MYCN* amplification (absolute copy number, > 10; Supplemental Fig 1A). Of these, 4 are considered *RB1* proficient, with 3 exhibiting RB1^+/+^ and 1 previously characterized as RB1^–/+^ by clinical diagnostics because of a germline insertion. No additional somatic variants were identified either by clinical diagnostics or whole exome sequencing in this tumor sample. One *MYCN*-amplified tumor displayed complete *RB1* loss (*RB1*^–/–^), caused by a germline frameshift on exon 5 followed by loss of heterozygosity. This study aimed to establish whether the high-level *MYCN*-amplified, *RB1*-proficient (*MYCN*^A^ *RB1*^PRO^) retinoblastoma present in this cohort displays unique molecular features that discriminate them from the rest of the retinoblastoma genomic subtypes.

### *MYCN*-Amplified *RB1*-Proficient Retinoblastomas Possess Significantly Different Gene Expression Patterns Compared with Other Retinoblastoma Subtypes

**Figure 1:**
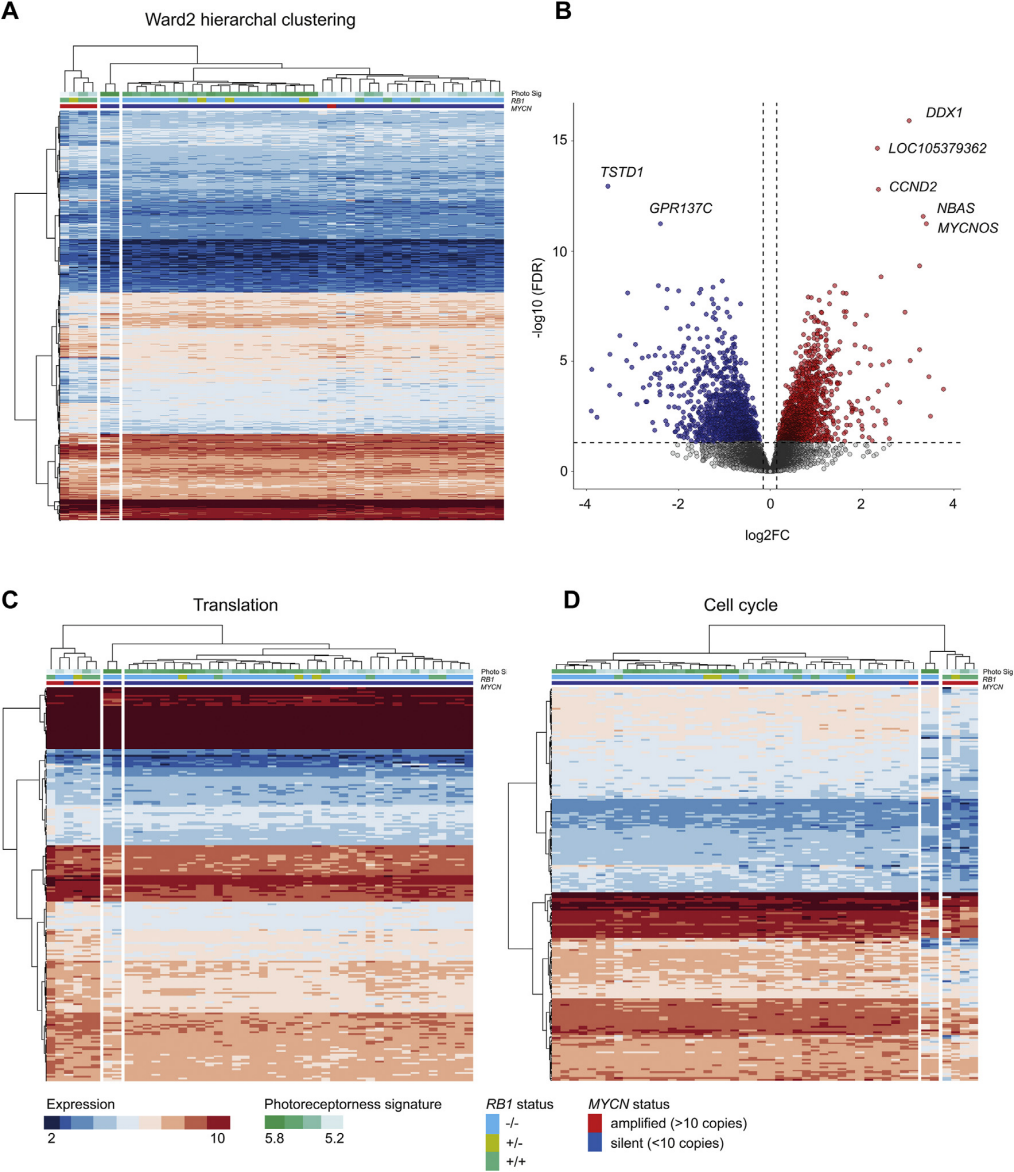
Differential expression analysis between *MYCN*-amplified *RB1*-proficient retinoblastoma tumors and *MYCN*-silent and *RB1*-deficient retinoblastoma tumors. *MYCN*-amplified, *RB1*-proficient retinoblastoma tumors display significantly different gene expression patterns compared with the *MYCN*-silent and *RB1*-deficient samples. **A**, Heatmap showing hierarchal clustering (WARD2) findings. The relationship between samples based on the 3155 differentially expressed genes in *MYCN*^A^*RB1*^PRO^ retinoblastomas is depicted. Rows represent genes. Columns represent samples. Expression colors are adjusted by the level of expression. The *MYCN* and *RB1* status and samples’ photoreceptorness scores are annotated beneath the dendrogram. The *MYCN*^A^*RB1*^PRO^ tumors form their own distinct cluster, departing from other retinoblastoma tumors irrelevant of photoreceptorness background. **B**, Volcano plot showing the differentially expressed genes in *MYCN*^A^*RB1*^PRO^ retinoblastomas. Top *MYCN*-amplified upregulated genes are associated with chromosome 2 amplification. The most significantly downregulated gene in *MYCN*^A^*RB1*^PRO^ samples, *TSTD1*, functions in hydrogen-sulfide metabolism. **C**, Heatmap showing hierarchical clustering (WARD2) visualizing the expression of 229 ribosomal RNA processing and translation initiation-associated genes significantly upregulated in *MYCN*^A^*RB1*^PRO^ samples. The high-level *MYCN*-amplified tumors cluster together independent of *RB1* genotypic background or photoreceptorness levels. **D**, Heatmap showing hierarchical clustering (WARD2) for the expression of 220 cell cycle-related genes significantly downregulated in *MYCN*^A^*RB1*^PRO^ samples. *MYCN*-amplified samples with *RB1*-proficient background cluster together. Two *RB1*-null *MYCN*-silent samples indicate a closer relationship to *MYCN*^A^*RB1*^PRO^ samples and cluster in the adjacent subbranch. Expression values as normalized log_2_ robust multiarray average: upregulation, log_2_ fold change (FC) > 0 and adjusted *P* < 0.05; downregulation, log_2_ FC < 0 and adjusted *P* < 0.05. FDR = false dscovery rate.

To observe how different genomic backgrounds concerning *MYCN* amplification and *RB1* mutations may influence retinoblastoma tumors’ transcriptomic relationships, hierarchal WARD2 clustering based on RMA-normalized expression values was performed (Supplemental Fig 1B). Hierarchal clustering resulted in 2 main branches. The *MYCN*^A^ *RB1*^PRO^ tumors formed their own separate subbranch within branch 1. The tumor sample with the *MYCN*-amplified *RB1*-deficient (*MYCN*^A^ *RB1*^–/–^) background clustered within another subbranch in branch 1 and separated from *MYCN*^A^ *RB1*^PRO^ tumors. A previous study from our laboratory showed that branch 1 encompasses 2753 downregulated genes compared with branch 2 samples. These genes were collectively coined as photoreceptorness gene signatures and comprised genes functionally associated with visual perception. Based on the averaged expression of this gene set, each sample was assigned a photoreceptorness score. *MYCN-*amplified tumors together with other retinoblastoma tumors in branch 1 belonged to the category of samples with a continuously lower photoreceptorness score (Supplemental Fig 1B). To identify gene-expression patterns specifically for *MYCN*^A^ *RB1*^PRO^ tumors, differential expression analysis comparing *MYCN*^A^ *RB1*^PRO^ (n = 4) to the rest of retinoblastoma tumors (n = 43) was performed (Fig 1, Appendix 2). In total, 3155 differentially expressed genes were detected with 1407 downregulated (log_2_ FC < 0; adjusted *P* < 0.05) and 1748 upregulated (log_2_ FC < 0; adjusted *P* < 0.05) *MYCN*^A^ *RB1*^PRO^ samples. Hierarchical clustering showed a clear distinction between *MYCN*^A^ *RB1*^PRO^ tumors and the rest of the samples for the expression of these genes. This was independent of photoreceptorness score (Fig 1A). Moreover, *MYCN*^A^ *RB1*^PRO^ samples indicated an overall distinctive expression pattern compared with the *MYCN*^A^ *RB1*^–/–^ sample. The top upregulated genes in *MYCN*^A^ *RB1*^PRO^ were related to chromosome 2 amplification, most significantly the *DDX1*, *NBAS*, and *MYCNOS* genes. *TSTD1*, a hydrogen-sulfide metabolism-associated gene, and the MTORC1-related *GPR137C* gene were the top downregulated genes in *MYCN*^A^ *RB1*^PRO^ tumors (Fig 1B). Collectively, the expression analysis revealed that the *MYCN*^A^ *RB1*^PRO^ molecular background correlates with significantly distinct transcriptomic signatures that discriminates this retinoblastoma subgroup from other retinoblastoma primary tumors regardless of photoreceptorness signature.

### Gene Ontology Analysis Reveals an Upregulation of Genes Involved in Translation and Downregulation of Cell Cycle-Related Genes in *MYCN*^A^*RB1*^PRO^ Tumors

To annotate the differentially expressed genes in *MYCN*^A^ *RB1*^PRO^ retinoblastoma functionally, gene ontology analysis was performed using ToppGene. Functional enrichment analysis for *MYCN*^A^ *RB1*^PRO^*-*upregulated genes resulted in multiple overlapping biological processes involved with translation, such as ribosomal RNA processing and translational initiation and elongation (Appendix 3). This group of translation-associated genes collectively consisted of 229 genes (Appendix 4). Hierarchical clustering based on the expression of the translation-related genes resulted in separate branch for *MYCN*-amplified tumors, including the *MYCN*^A^ *RB1*^–/–^ tumor (Fig 1C). Functional annotation based on downregulated genes in *MYCN*^A^ *RB1*^PRO^ identified several overlapping biological processes associated with the cell cycle (Appendix 3). This cell cycle-associated category comprised 220 genes (Appendix 4). Hierarchical clustering showed a separation of the *MYCN*^A^ *RB1*^PRO^ genes from the rest of cohort with the exception of 2 *MYCN-*silent *RB1*^–/–^ samples that cluster in a subbranch adjacent to the *MYCN*^A^ *RB1*^PRO^ branch (Fig 1D). Furthermore, the cell cycle gene group comprises a diverse array of functional categories such as DNA repair and replication, negative regulation of cell cycle phase transition (includes 6 cyclin kinase inhibitors), regulation of G2/M mitotic cell cycle transition, and sister chromatid segregation. Overall, functional enrichment analysis showed that the differential transcriptomic signatures of *MYCN*^A^ *RB1*^PRO^ are highly functionally relevant because they are associated with major and general biological processes.

### Low Expression of Both Cone and Rod Photoreceptor Markers in *MYCN*-Amplified *RB1*-Proficient Retinoblastomas

Looking further into the importance of retinal markers in *MYCN*^A^ *RB1*^PRO^ retinoblastomas, we performed clustering analysis on the entire cohort specifically for genes expressed in cone and rod photoreceptors, Müller glia, horizontal, bipolar, amacrine astrocytes, microglia, and ganglion cells (Supplemental Fig 2). This resulted in a scattered clustering of *MYCN*^A^ *RB1*^PRO^ samples within different subbranches. As the clustering showed, an overall tendency exists for the *MYCN*^A^ *RB1*^PRO^ samples to reside relatively closer to retinoblastoma tumors with lower photoreceptorness scores, which infers relatively lower expression of retina markers associated with visual perception (Supplemental Fig 2). Overall, the analysis did not identify a unique expression pattern for a specific set of retina markers that widely separate *MYCN*^A^ *RB1*^PRO^ from other retinoblastomas.

### At the Methylation Level, *MYCN*-Amplified *RB1*-Proficient Retinoblastomas Are Distinct from *MYCN*-Silent Retinoblastomas

**Figure 2:**
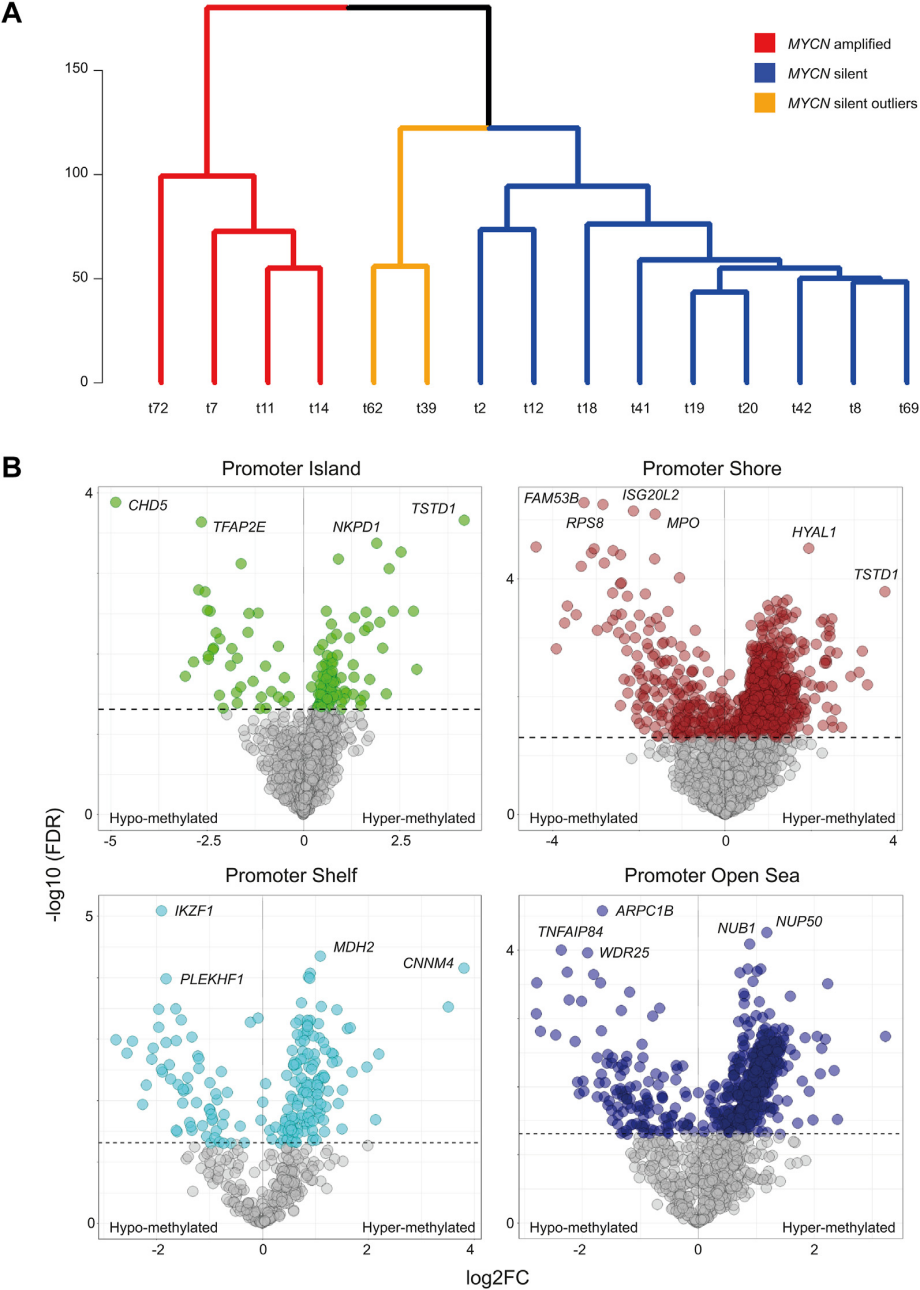
Differential methylation analysis between a group of 4 high-level *MYCN*-amplified and a group of 11 *MYCN*-silent retinoblastomas. *MYCN* tumors possess distinct methylation patterns compared with non–*MYCN*-amplified retinoblastomas. **A**, Dendrogram showing the hierarchical clustering relationships of the retinoblastoma samples. Based on the WARD2 clustering method, *MYCN*-amplified tumors display distinct methylation patterns compared with *MYCN*-silent retinoblastomas. **B**, Volcano plots depicting the differentially methylated genes at various promoter methylation sites between *MYCN*-amplified and *MYCN*-silent samples. All sites indicate significantly differentially methylated genes between *MYCN*-amplified and *MYCN*-silent samples. Methylated signal is quantile-normalized β values: hypermethylated, log_2_ fold change (FC) > 0 and adjusted *P* < 0.05; hypomethylated, log_2_ FC < 0 and adjusted *P* < 0.05). FDR = false discovery rate.

Of the 47 samples in the retinoblastoma cohort, methylation profiles for 15 samples, including the 4 *MYCN*^A^ *RB1*^PRO^ samples, were available. These profiles were examined to explore the significant epigenetic differences between *MYCN*-amplified and *MYCN*-silent tumors. In addition, the methylation and expression profile of the 15 samples were integrated to identify the most significant methylation-driven gene expression in MYCN tumors. We subsequently cross-examined the reproducibility of these findings (n = 15) in the entire retinoblastoma cohort (n = 47) and publicly available data. Unsupervised hierarchical clustering of *MYCN*-amplified and *MYCN*-silent tumors based on normalized methylated signals (β value) resulted in complete separation of *MYCN*-amplified samples from *MYCN*-silent samples (Fig 2A). Differential methylation analysis was performed to assess the amplitude of methylation differences between the two retinoblastoma groups (Appendix 5). Because promoter site methylations have more effect on gene expression, the downstream analysis mainly focused on differentially methylated genes in this region. Moreover, because different methyl sites in relationship to the CpG island may impact gene expression differently, the differentially methylated genes between *MYCN*-amplified and *MYCN*-silent samples for promoter island, shore, shelf, and open sea were modeled and evaluated separately (Fig 2B). For all the different methylation sites, many genes showed significant differential methylation between *MYCN*-amplified and *MYCN*-silent samples, but promoter shore and promoter open sea were the most densely differentially methylated regions between *MYCN*-amplified and *MYCN*-silent tumors. Altogether, methylation analysis, in line with transcriptomic profiling, showed unique methylation patterns in *MYCN*^A^ *RB1*^PRO^ tumors that distinguishes them from other retinoblastomas.

### Promotor Hypermethylation Is the Main Driver of Methylation-Specific RNA Expression Changes in *MYCN*-Amplified Retinoblastomas

**Figure 3:**
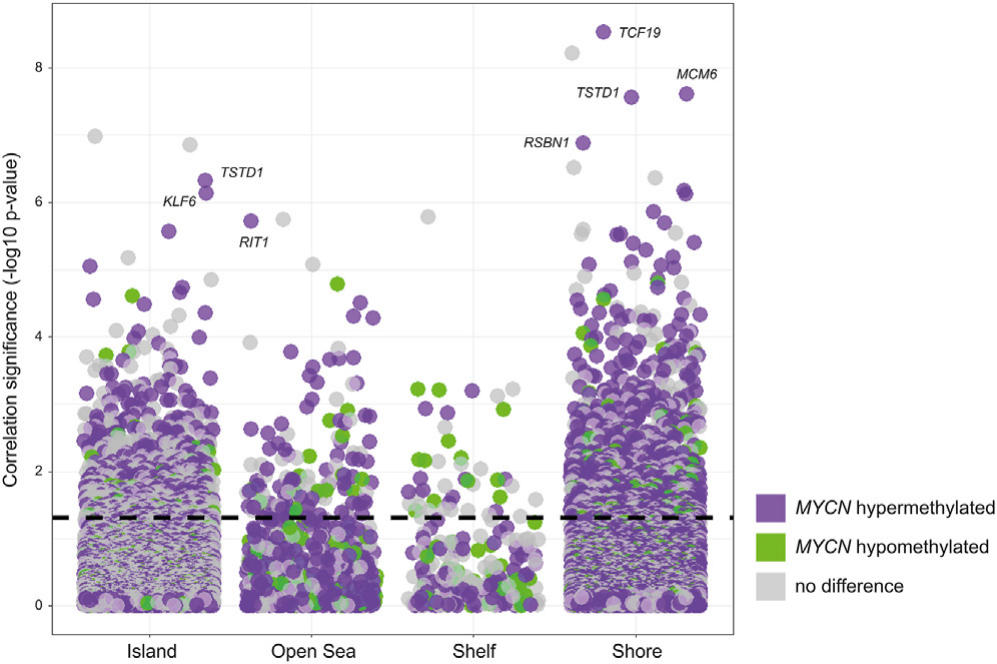
Promoter methylation-expression correlation analysis. Methylation status significantly influences gene expression in *MYCN*-amplified samples. Correlation jitter plot presenting the significance of methylation-expression correlations across the retinoblastoma panel, based on normalized methylated signal β values and the expression log_2_ robust multiarray averages. Correlations with *R* < 0 and *q* < 0.05 were considered significant. The y-axis presents correlation significance as the minus log_10_-transformed *q* value. The x-axis presents the methylation region. In *MYCN*-amplified tumors, hypermethylation has more overall significant influence on gene expression than hypomethylation. *TCF19* and *TSTD1* are the most significant methylation expression-correlated genes in *MYCN*-amplified tumors.

To identify genes whose expression levels were significantly determined by methylation, Pearson correlation analysis between gene-level methylated signals (β values) and normalized gene expression levels was performed (Fig 3, Appendix 6). Correlations with *R* < 0 and *q* < 0.05 were deemed significant. Promoter methylation in shores followed by islands correlated most significantly with RNA expression levels. However, methylation levels at shelfs and open sea sites showed less effect on gene expression. Based on the correlation analysis, the hypermethylation in *MYCN*-amplified samples versus *MYCN*-silent samples was the most predominant source of mRNA expression changes, with many genes hypermethylated in *MYCN*-amplified tumors showing significant methylation-expression correlations. The *MYCN*-hypermethylated genes *TCF19*, *MCM6*, *TSTD1*, *RSBN1*, *KLF6*, and *RIT1* were the most significant methylation-driven genes. Overall, it was shown that the hypermethylation observed in high *MYCN*-amplified *RB1*-proficient retinoblastomas has significant influence on global gene expression in this tumor subtype.

### *MYCN*-Amplified *RB1*-Proficient Retinoblastomas Express a Unique Set of Hypermethylation-Driven Genes That Distinguishes Them from Other Retinoblastomas

To identify hypermethylated and hypomethylated genes that correlated with significant differential expression between *MYCN*^A^ *RB1*^PRO^ retinoblastomas and the rest of the cohort, the differential expression, methylation, and correlation results were integrated (Supplemental Fig 3). This facilitated the identification of genes in which hypermethylation in *MYCN*^A^ *RB1*^PRO^ retinoblastoma (log_2_ FC > 0; adjusted *P* < 0.05) strongly correlated with expression (*R* < 0; *q* < 0.05) and subsequently resulted in significant downregulation in *MYCN*^A^ *RB1*^PRO^ samples (log_2_ FC < 0; adjusted *P* < 0.05). The data integration was also performed in the opposite direction to determine hypomethylation-driven upregulated genes in *MYCN*^A^ *RB1*^PRO^ retinoblastomas compared with the rest of the cohort.

The integrated analysis identified 79 overlapping genes that showed hypermethylation on promoter shore or island regions and that were significantly downregulated in *MYCN*^A^ *RB1*^PRO^ retinoblastomas compared with *MYCN*-silent retinoblastomas (Supplemental Fig 4). Considerably fewer shelf and open sea hypermethylated genes showed significant downregulation in *MYCN*-amplified retinoblastomas. Nine genes were identified that showed hypomethylation and were significantly upregulated in *MYCN*^A^ *RB1*^PRO^ retinoblastomas. Our data show that hypermethylation in *MYCN*-amplified samples is the most important epigenetic event inducing gene expression differences between *MYCN*^A^ *RB1*^PRO^ retinoblastoma and the other retinoblastomas.

**Figure 4:**
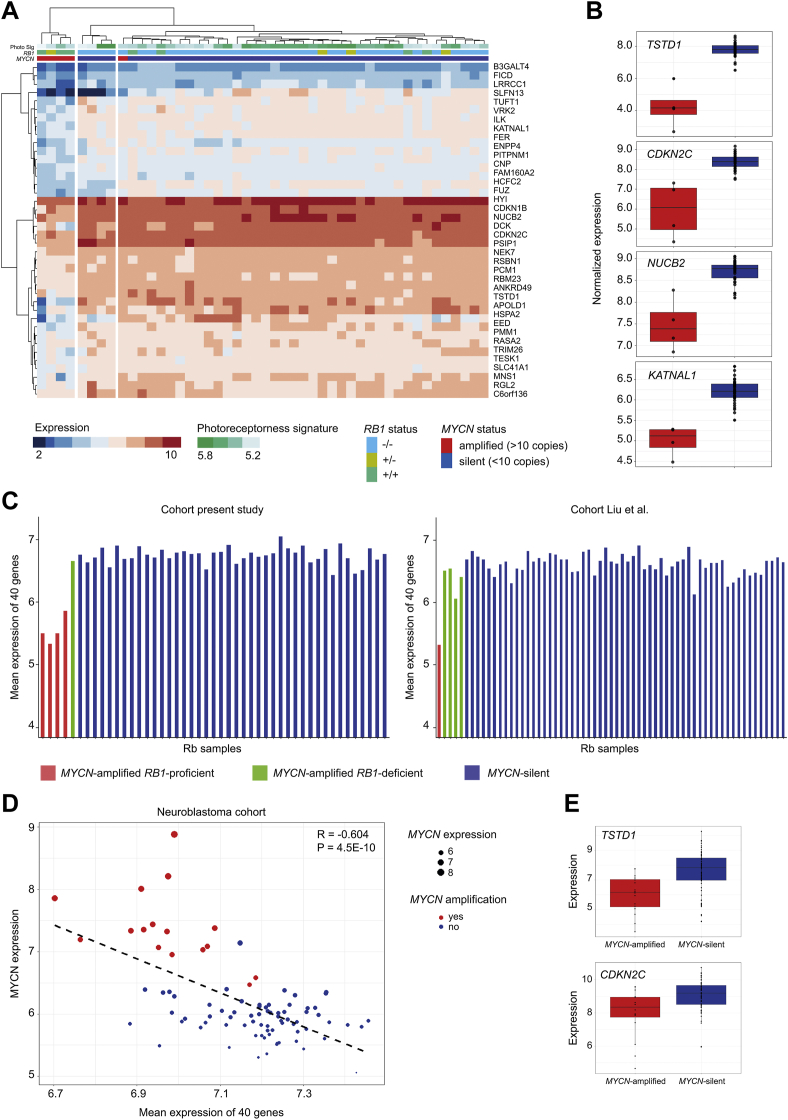
Methylation-driven differentially expressed genes in *MYCN*-amplified *RB1-*proficient retinoblastomas. *MYCN*-driven, *RB1*-proficient tumors display unique methylation-derived gene expression signatures that globally discriminate them from other retinoblastoma tumors. **A**, Heatmap showing hierarchal clustering for 40 genes that are hypermethylated (log_2_ fold change [FC] > 0; adjusted *P* < 0.05), downregulated (log_2_ FC < 0; adjusted *P* < 0.05), and indicate significant methylation-expression correlation (*R* < 0; *q* < 0.05) in *MYCN*^A^*RB1*^PRO^ retinoblastomas. The distinct expression of the 40 genes in *MYCN*^A^*RB1*^PRO^ samples primarily identified within a subset of the cohort (n = 15) is also reproducible in the entirety of the local retinoblastoma cohort (n = 47). For the expression of these genes, *MYCN*^A^*RB1*^PRO^ retinoblastomas (n = 4) show clear-cut separation from the rest of the retinoblastoma cohort (n = 47). **B**, Box-dot plots demonstrating normalized expression values of the top 4 hypermethylation-driven downregulated genes in *MYCN*^A^*RB1*^PRO^ versus the rest of the retinoblastoma cohort. **C**, Bar charts showing averaged expression of the 40 hypermethylated-driven genes discriminating *MYCN*^A^*RB1*^PRO^ retinoblastomas from other retinoblastoma subtypes: the current study (left) and public domain expression data (Liu et al^10^) including 62 retinoblastoma tumors (right). Red indicates *MYCN*-amplified *RB1*-proficient retinoblastomas. Green indicates *MYCN*-amplified *RB1*-deficient retinoblastomas. Blue indicates *MYCN*-silent retinoblastomas. Expression values are as normalized log_2_ robust multiarray average. **D**, Scatterplot depicting the association between *MYCN* expression and the mean expression of the 40 *MYCN*^A^*RB1*^PRO^ hypermethylation-driven downregulated genes in neuroblastoma (Pearson method). *MYCN*-amplified samples appear in red and *MYCN-*silent samples appear in blue. The dot sizes are adjusted by the expression level of *MYCN*. *MYCN* expression shows a significant negative correlation with the expression of the 40 signature genes (*R* = –0.604; *P* = 4.5 × 10^–10^). **E**, Box-dot plots for the normalized expression values of *TSTD1* and *CDKN2A* within *MYCN*-amplified and silent neuroblastomas. Rb = retinoblastoma.

To determine whether these results were also reproducible in a broader context, the findings were cross-validated using RNA expression data available from the entire retinoblastoma cohort (n = 47). Hierarchical clustering for the expression values of the 79 hypermethylated genes, but not for the hypomethylated genes, resulted in clear distinction in expression between *MYCN*^A^ *RB1*^PRO^ and the rest of the retinoblastomas (Supplemental Fig 5). The unique expression pattern was also reflected in expression-fold changes between *MYCN*^A^ *RB1*^PRO^ and the rest of the retinoblastomas (Appendix 7). All 79 genes showed a downregulation in *MYCN*^A^ *RB1*^PRO^, with 74 being statistically significant. Although expression of all these genes was significantly decreased in *MYCN*^A^ *RB1*^PRO^ tumors, their expression was not clear-cut and may be partially influenced by the relatively low photoreceptorness score observed in this *MYCN*^A^ *RB1*^PRO^ retinoblastoma subgroup. Thus, to extract the most significant expression signatures exclusive to *MYCN*^A^ *RB1*^PRO^ retinoblastomas, we performed 3 additional differential expression analysis. Analyses 1 and 2 were between *MYCN*^A^ *RB1*^PRO^ retinoblastomas and the 2 main retinoblastoma subbranches (adjusted *P* < 0.05, *MYCN* vs. clusters 1 and 2), whereas analysis 3 was between branches 1 and 2 to filter out genes that were differentially expressed between the two (adjusted *P* > 0.1, cluster 2 vs. cluster 1; Supplemental Figs 3 and 5, Appendix 8). Subsequently, a refined list of 40 genes was produced showing downregulation most exclusively in *MYCN*^A^ *RB1*^PRO^ retinoblastomas (Appendix 9). This was confirmed by hierarchal clustering, which showed a clear separation between *MYCN*^A^ *RB1*^PRO^ and the rest of the retinoblastoma cohort (Fig 4A). *TSTD1*, *CDKN2C*, *NUCB2*, and *KATNAL1* showed the most significant *MYCN*^A^ *RB1*^PRO^-specific downregulated expression pattern among the hypermethylated genes in this retinoblastoma subtype (Fig 4B). In addition, averaged expression of the 40 genes displayed a clear distinction between different retinoblastoma groups. *MYCN*^A^ *RB1*^PRO^ retinoblastomas showed a clear downregulation of these 40 genes, while the *MYCN*^A^ *RB1*^DEF^ retinoblastoma sample in this cohort showed a similar expression signature as the *MYCN*-silent retinoblastomas (Fig 4C, left). Our findings were also validated in an independent previously published retinoblastoma cohort by Liu et al^10^ (Fig 4C, right; Supplemental Fig 6), where the *MYCN*^A^ *RB1*^PRO^ retinoblastoma showed an average lower expression of this gene panel in comparison with the rest of the retinoblastoma cohort. Overall, the integrated analysis revealed that the hypermethylation pattern in *MYCN*^A^ *RB1*^PRO^ tumors enforces significant downregulation of a gene set in these tumors that discriminates the *MYCN*^A^ *RB1*^PRO^ tumor from all other retinoblastoma subtypes.

### The Hypermethylation-Driven, Downregulated Genes in *MYCN*^A^*RB1*^PRO^ Tumors Also Show Overall Lower Expression in *MYCN*-Amplified Neuroblastoma

Furthermore, because *MYCN* amplification is also a common somatic event in neuroblastoma, we examined the behavior of the signature genes in a public data set derived from 88 neuroblastoma tumors, with 16 harboring a *MYCN* amplification (Fig 4D, E). Differential expression analysis verified a significantly higher expression of *MYCN* in the *MYCN-*amplified neuroblastomas. Subsequently, we performed a Pearson correlation analysis between the expression of *MYCN* and the mean expression of the signature genes. The analysis showed a significant negative correlation between mean expression of the *MYCN*^*A*^ *RB1*^*PRO*^ signature genes and the expression of *MYCN* (*R* = –0.604; *P* = 4.5 × 10^–10^; Fig 4D). We also examined the expression of the 40 genes between *MYCN*-amplified and *MYCN*-silent neuroblastoma to identify the genes contributing most significantly to the observed correlation. A total of 33 of the 40 genes showed a negative-fold change in expression in *MYCN*-amplified neuroblastomas compared with *MYCN*-silent samples, with 11 being significant by the adjusted *P* value of < 0.05 (Appendix 10). These included the top *MYCN*^*A*^ *RB1*^*PRO*^ downregulated *TSTD1* and *CDKN2C* genes, which also showed a significantly lowered expression in *MYCN-*amplified compared with the *MYCN*-silent neuroblastomas (Fig 4E). Overall, the expression patterns observed in neuroblastoma *MYCN-*amplified samples were in line with the expression of these genes in *MYCN*^*A*^ *RB1*^*PRO*^, suggesting a broader implication for the observed signatures in *MYCN*-amplified cancers beyond retinoblastoma.

### The Hypermethylation-Driven, Downregulated Genes in *MYCN*^A^*RB1*^PRO^ Tumors Are Interactionally Enriched and Linked to a Number of Pathways Including G1 to S-Phase Transition

**Figure 5:**
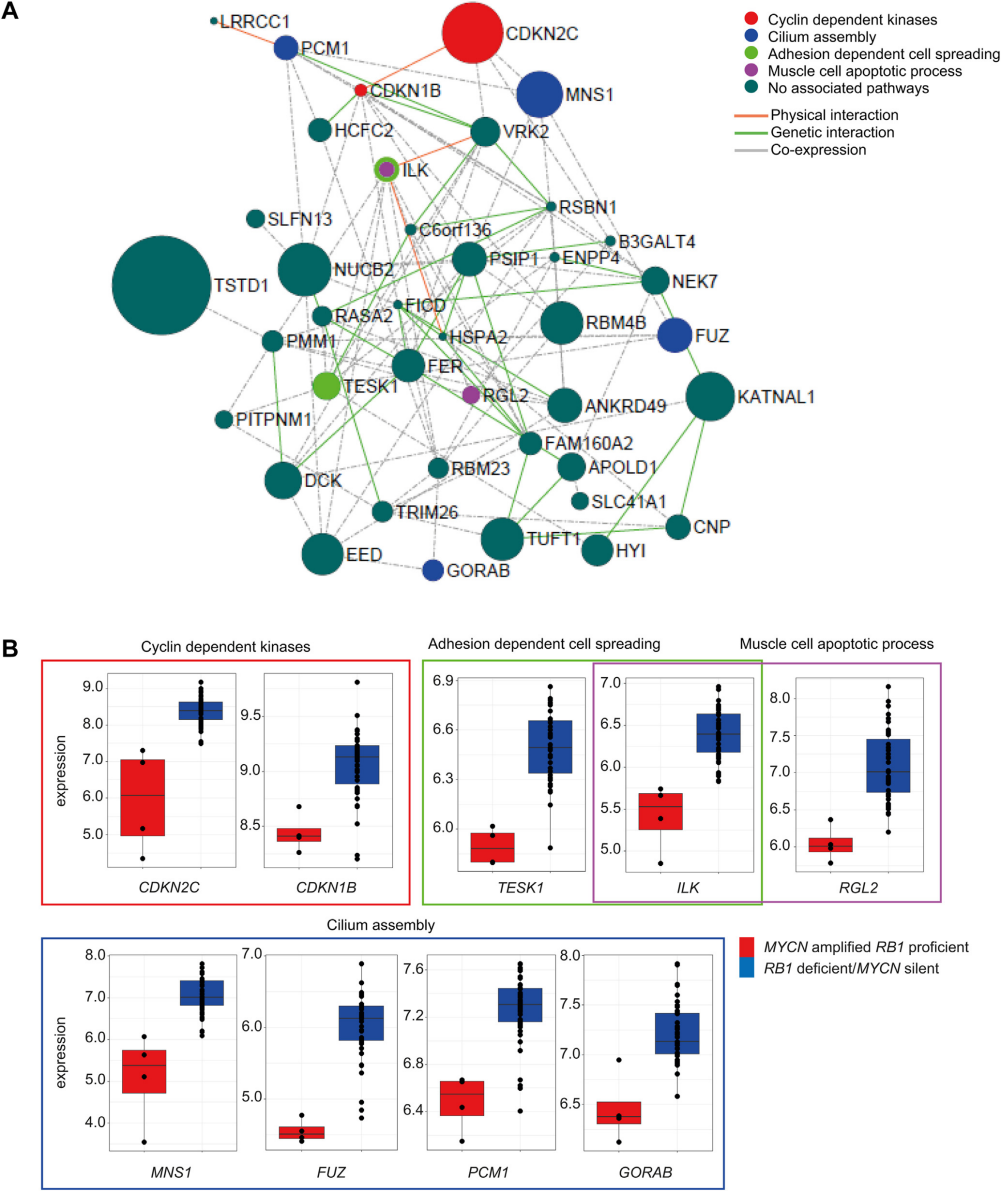
Functional and gene–gene interaction analysis for methylation-driven differentially expressed genes in *MYCN*-amplified tumors. **A**, Interaction network with functional annotation depicting the interaction between genes as well as their pathway enrichment. Interaction network analysis was conducted by GeneMANIA and pathway analysis was conducted by ClueGo. Nodes represent genes. Edges represent interaction. Node sizes are adjusted based on the significance of the specific downregulation in *MYCN*^A^*RB1*^PRO^ tumors: largest node, –log_10_ adjusted *P* = 11.5; smallest node, –log_10_ adjusted *P* = 2.5). Node colors are adjusted based on the functional group they belong to. Edge’s types are drawn based on the type of interaction. **B**, Box plots showing for the expression of the downregulated pathways in *MYCN*^A^*RB1*^PRO^ tumors. Expression values as normalized log_2_ robust multiarray average.

Gene–gene interaction and functional network analysis was performed using GeneMANIA and ClueGo, respectively, for the 40 *MYCN*^A^ *RB1*^PRO^ hypermethylation-driven downregulated genes. Network analysis detected several genetic and physical interactions between these genes (Fig 5A). Pathway analysis showed that some of these genes were enriched for functionally relevant processes. These were cyclin-dependent kinase inhibitor activity (*CDKN2C* and *CDKN1B*), muscle cell apoptotic process (*ILK* and *RGL2*), adhesion-dependent cell spreading (*ILK* and *TESK1*), and nonmotile cilium assembly (*GORAB*, *PCM1*, *FUZ*, and *MNS1*; Fig 5B). In summary, network and pathway analysis identified a set of interacting and functionally related genes that, based on their methylation-driven expression pattern, significantly discriminate *MYCN*^A^ *RB1*^PRO^ tumors from the rest of retinoblastomas.

## Discussion

In this study, we explored unique transcriptomic and epigenomic features of *MYCN*-amplified *RB1*-proficient retinoblastomas compared with other retinoblastomas. The genome-wide mRNA expression and methylation analysis identified large numbers of differentially expressed and methylated genes. Using an integrated approach, we identified a set of hypermethylation-driven downregulated genes in *MYCN*-amplified *RB1*-proficient tumors. The *MYCN*-amplified *RB1*-proficient specific downregulated genes were also validated analyzing an independent cohort, inferring the universality of the findings. Moreover, we observed a downregulated pattern for the expression of at least a subset of these genes in *MYCN*-amplified neuroblastoma compared with *MYCN*-silent neuroblastoma. Overall, the results showed that *MYCN*-amplified *RB1*-proficient retinoblastomas do possess distinct molecular signatures that diverge them from *MYCN-*silent or *RB1*-null retinoblastoma, or both. This in turn may point to differential biological or oncogenic dynamics within this rare retinoblastoma subtype, which is already clinically reflected in the earlier age at diagnosis compared with that of classical *RB1*^–/–^ retinoblastomas.^6^ Based on the neuroblastoma data, this distinct property may also have implications beyond retinoblastoma and may be linked to the general biological features of pediatric cancers harboring *MYCN* amplification.

A previous transcriptomic-based analysis revealed 2 major subgroups within retinoblastoma, interacting in a continuous fashion.^9^ A recent multiomics classifications study also further postulated the existence of 2 major retinoblastoma groups.^10^ The present analysis reconfirmed the clustering of *MYCN*-amplified *RB1*-proficient wild-type tumors within a retinoblastoma subbranch characterized by lower photoreceptorness. However, it also extracted a large set of differentially expressed genes in *MYCN*-amplified *RB1*-proficient samples. This set clearly distinguished the subgroup from other retinoblastomas, independently of their photoreceptorness grade. It is suggested that these gene signatures infer unique molecular characteristics that are possessed by these rare tumors. We showed that genes upregulated in *MYCN-*amplified *RB1*-proficient retinoblastomas were significantly enriched for translation and mRNA synthesis processes, whereas downregulated genes were enriched for cell cycle regulation pathways. The upregulation of translation and mRNA synthesis in *MYCN*-amplified *RB1*-proficient retinoblastomas may be the direct outcome of *MYCN* overexpression. It is well known that the *MYCN* oncogene significantly impacts on translation via boosting ribosomal biogenesis and mRNA translation.^20^^,^^21^ *MYCN* overexpression also influences the cell cycle.^22^^,^^23^ The fact that we find downregulation of genes that are important for proliferation in *MYCN*-amplified *RB1*-proficient retinoblastoma may initially seem counterintuitive. However, it is noted that not all cell cycle-related genes on the list influence cell cycle dynamics in a similar fashion, and the downregulation of different cell cycle genes or subclasses may impose different and at times opposing effects on cell cycle progression. In fact, the downregulation of cell cycle G1 to S-phase transition genes such as cyclin kinase inhibitors may promote further proliferation, rather than inhibition, in *MYCN*-amplified *RB1*-proficient retinoblastoma. Our analysis indicated, in concordance with the literature, a systematic downregulation of 6 cytokine kinase inhibitors.^22^ This can potentially induce the acceleration of cell cycle transition through G1.^24^ These results are in line with and complement the upregulation of *CCND2* observed in the *MYCN*-amplified *RB1*-proficient retinoblastomas. *CCND2* is an important regulator of cell cycle and it facilitates G1 to S-phase transition. The hyperactivity of *CCND2* has been associated with malignancy, and it promotes proliferation in cancer cell lines.^25^ In line with this, one of the most striking and statistically significant findings in the study was the hypermethylation-driven downregulation of *CDKN2C.* This may imply a selective pressure for the repression of the cyclin-dependent kinase inhibitors activity within the *MYCN*-amplified *RB1*-proficient genomic background. *CDKN2C* in particular retained the most consistent and significantly declined mRNA expression in *MYCN*-amplified *RB1-*proficient tumors. *CDKN2C* blocks cell cycle progression via its interaction with CDK4 and CDK6. Consequently, it has been shown that the declined *CDKN2C* activity can lead to dysregulated S-phase cell cycle progression, which functionally resembles a *RB1* null-like cell cycle behavior.^26^^,^^27^ *CDKN2C* repression has been reported in multiple cancers, including melanoma, esophageal squamous cell carcinoma, and pituitary adenomas.^28^, ^29^, ^30^ Furthermore, a recent genomic study in leiomyosarcoma characterized by frequent *RB1*/*TP53* mutations identified a distinct class featuring wild-type *TP53*/*RB1* and homozygous deletion of *CDKN2C*.^31^ Consequently, it is hypothesized that the hypermethylation of *CDKN2C* in *MYCN*-amplified *RB1*-proficient retinoblastomas may be a distinct oncogenic event and it may potentially act as an alternative to *RB1* deactivation in dysregulating G1 and S-phase cell cycle progress. The analysis of *MYCN*-amplified versus *MYCN*-silent neuroblastoma also showed a significant downregulation of *CDKN2C* in *MYCN*-amplified samples, suggesting a potential, general oncogenic function for the lowered expression of *CDKN2C* for *MYCN*-amplified pediatric cancers.

In fact, the differences in molecular spectra of *MYCN*-amplified *RB1-*proficient retinoblastomas compared with other retinoblastoma subtypes were very well marked at the epigenomic level, which affected a molecular network beyond *CDKN2C.* Epigenetic alterations are well-known cancer events with versatile impact on various stages of carcinogenesis, ranging from disruption of genomic stability to silencing of tumor suppressors and regulatory elements.^32^^,^^33^ In addition, methylation patterns can potentially be used as biomarkers in tissue characterization, prognosis prediction, and adapting cancer treatment strategies.^34^ Differential methylation profiling identified a large number of significantly hypomethylated and hypermethylated genes in *MYCN-*amplified *RB1*-proficient wild-type tumors compared with *MYCN*-silent retinoblastomas. The integrated methylation expression analysis pointed to hypermethylation rather than hypomethylation as the main epigenetic influencer of gene expression differences between the 2 retinoblastoma subtypes. This was projected most notably in the case of 40 hypermethylated downregulated genes in *MYCN*-amplified *RB1-*proficient samples. The expression pattern of these genes clearly separates *MYCN*-amplified *RB1*-proficient tumors from other retinoblastomas, observed in both in-house and public-domain patient cohorts. The *MYCN*-amplified *RB1*-proficient hypermethylation-driven, differentially expressed genes are a functionally diverse set of genes, suggesting that hypermethylation in this tumor subtype is indeed not functionally unidirectional, and it may be modulating multiple aspects of the tumor biology. Next to cell cycle transition-related *CDKN2C*, the other top distinctly downregulated gene, *thiosulfate sulfurtransferase-like domain containing 1* (*TSTD1*), is of a radically different functional class. This gene, in fact, is poorly characterized; however, it is established to be involved in hydrogen sulfide metabolism by the means of S-sulfanylglutathione production.^35^, ^36^, ^37^ Hence, the very distinct and specifically lowered expression level of this gene potentially hints at the existence of differential metabolic dynamics within *MYCN*-amplified *RB1*-proficient retinoblastomas. In addition, cilia assembly was also identified as a potential differential regulated pathway in *MYCN*-amplified *RB1*-proficient retinoblastomas compared with the rest of the cohort. Cilia are sensory organelles that protrude from the cell surface into the extracellular space and are expressed on all nonhematologic cell types in the body. They are important for communication between cells and the tumor microenvironment and are associated with several important signaling pathways. The formation of cilia is linked to control of the cell cycle, where they occur in the G0 to early G1 phases and vanishes again in S or G2 phase. Because oncogenic signaling, for example by *MYCN*, stimulates proliferation, this can result in the loss or shortening of primary cilia.^38^

In conclusion, although *MYCN*-amplified *RB1*-proficient tumors reside within the molecular spectra of retinoblastoma, they still possess significant differences in expression and methylation patterns that distinguish them from the other retinoblastoma tumors. This may emphasize the importance of also implementing more targeted and gene set-oriented approaches when classifying and labeling tumor subtypes. Tumors, which may be interrelated globally, still may possess a subset of features that significantly diverge them from each other. This indeed is the trend we see in the case of *MYCN* tumors. The significantly distinct molecular components in *MYCN* tumors, such as upregulated translation processes or hypermethylated genes related to metabolism, cell cycle transition, or cilium assembly, may imply the presence of differential cellular and oncogenic dynamics unique to the *MYCN*-amplified *RB1*-proficient tumor subtype. The recently developed noninvasive methods for the detection of gene amplification in tumors, analyzing circulating free DNA present in aqueous humor of affected eyes,^39^ may be relevant in this context. It can be especially expected that the large *MYCN* amplifications, as present in the *MYCN*-amplified tumor, may be detected relatively more easily. Detection of *MYCN* amplification may aid the decision-making process regarding whether to enucleate an eye. Furthermore, it can be envisioned that the unique *MYCN* signature we discovered in these tumors may be explored in the future to adapt a more personalized and targeted approach when treating children with *MYCN*-amplified retinoblastoma.
