# Supplementary material for: High-Level MYCN-Amplified RB1-Proficient Retinoblastoma Tumors Retain Distinct Molecular Signatures
Source: Ophthalmol Sci. 2022 Jun 20;2(3):100188. doi: 10.1016/j.xops.2022.100188 (PMC9559112; doi:10.1016/j.xops.2022.100188)
Supplement: Supplemental Figures S1-S6 [file mmc1.pdf]

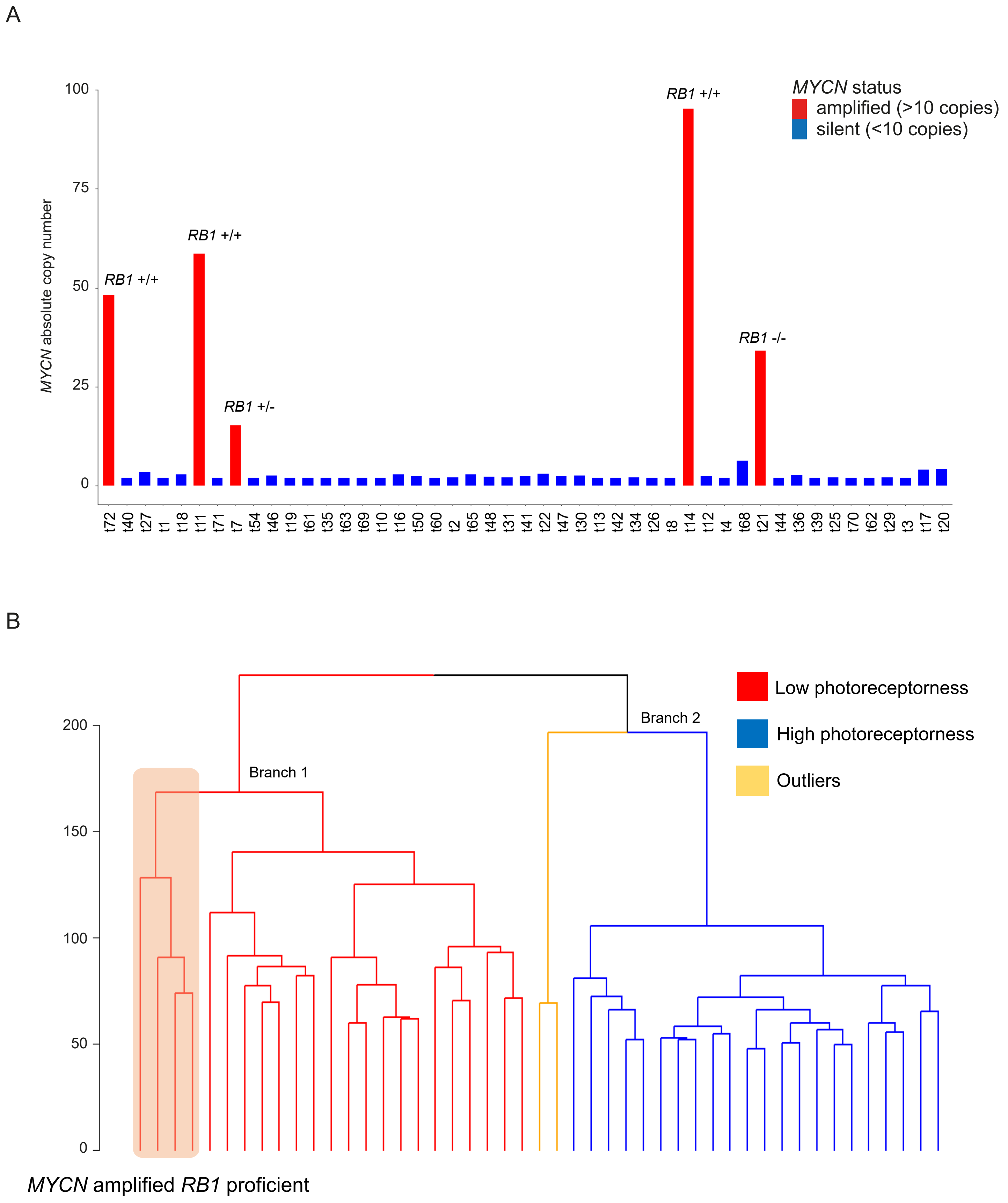

*

Supplementary figure 1. The retinoblastoma cohort. **A)** Bar chart presenting the copy number of *MYCN* in the retinoblastoma cohort (N=47). Y-axis presents absolute copy number. X-axis presents samples. Overall, 5 samples indicate high-level *MYCN* amplification (>10), with 4 out 5 being *RB1*-proficient. **B)** Dendrogram presenting the hierarchical relations between retinoblastoma samples based on RMA- normalized expression values by the WARD2 method. Clustering results in to two main branches with *MYCN*-amplified *RB1*-proficient samples residing in branch 1, previously had been described as low photoreceptorness sub-group.

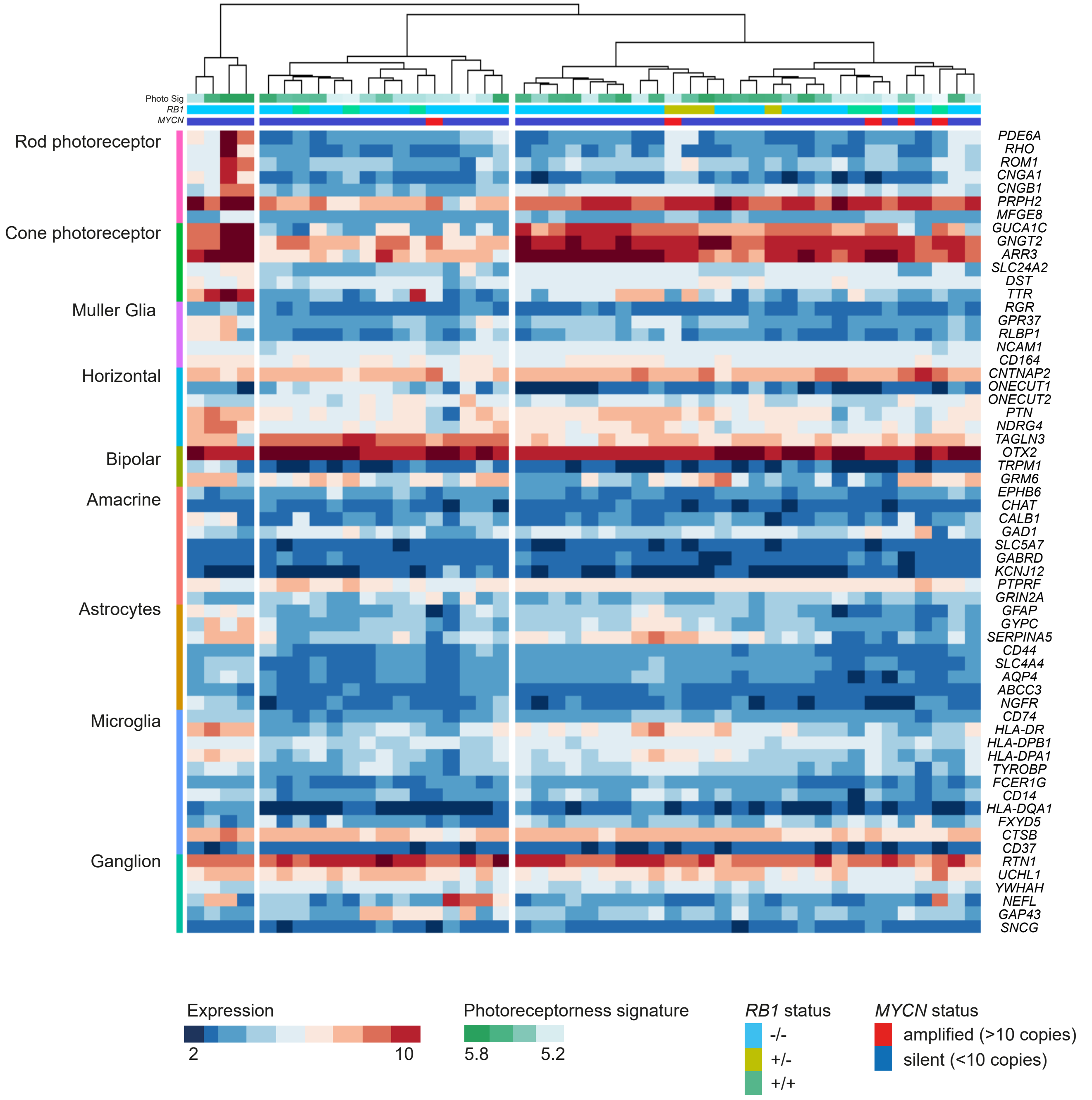

Supplementary figure 2. The mRNA expression of major retina markers in Retinoblastoma cohort. Hierarchal clustering (WARD2) with heat map depicting the relationship between samples based on the expression of major known retina markers.

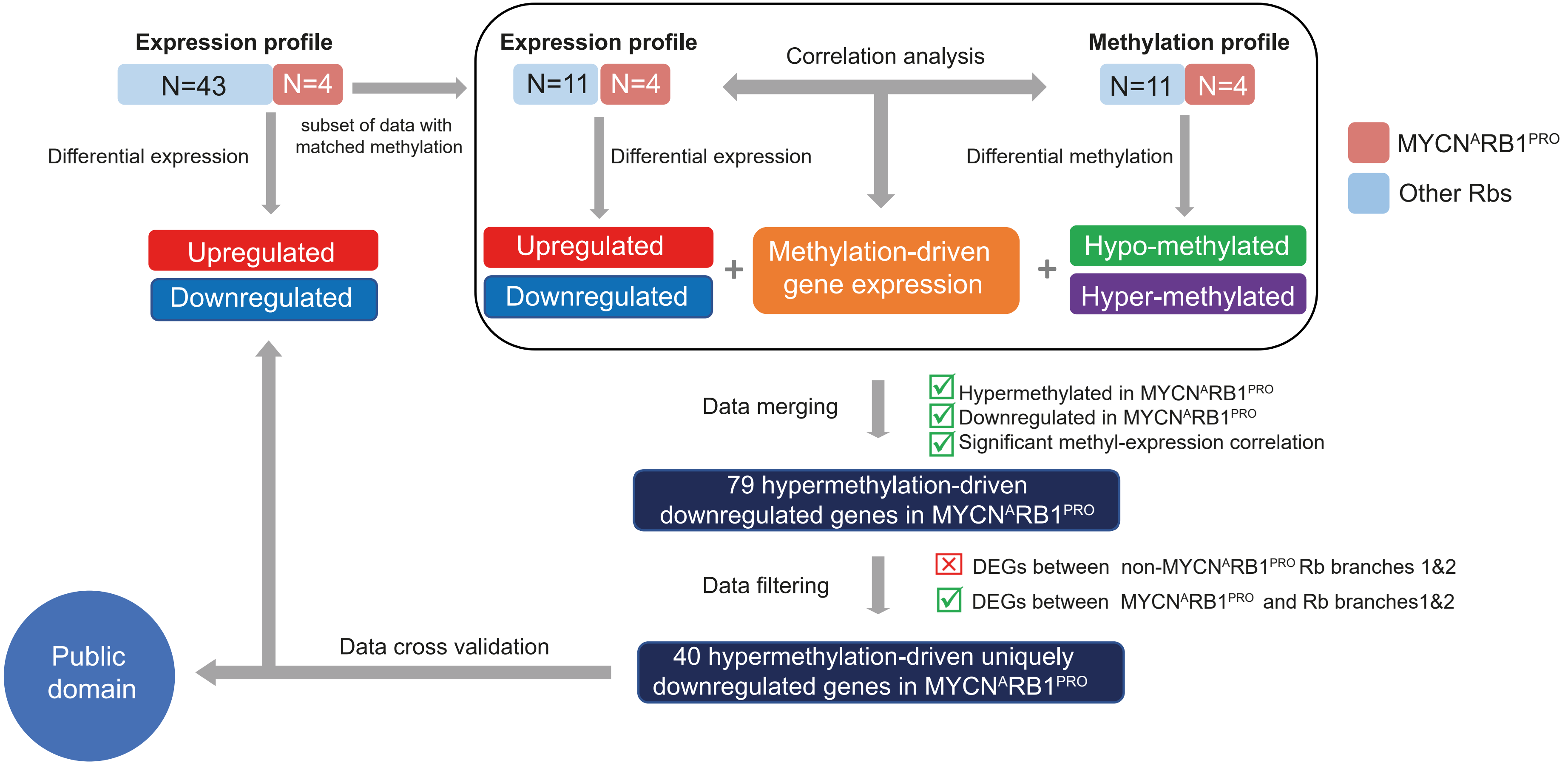

Supplementary figure 3. The workflow for the integrated expression-methylation analysis of retinoblastoma cohort. Upregulated: Log2FC > 0, Adjusted p.value < 0.05. Downregulated: Log2FC < 0, Adjusted p.value < 0.05. Hyper-methylated: Log2FC > 0, Adjusted p.value < 0.05. Hypo-methylated: Log2FC < 0, Adjusted p.value < 0.05. Significant Methyl-expression correlation: R < 0, q.value < 0.05. DEGs: Differentially Expressed Genes.

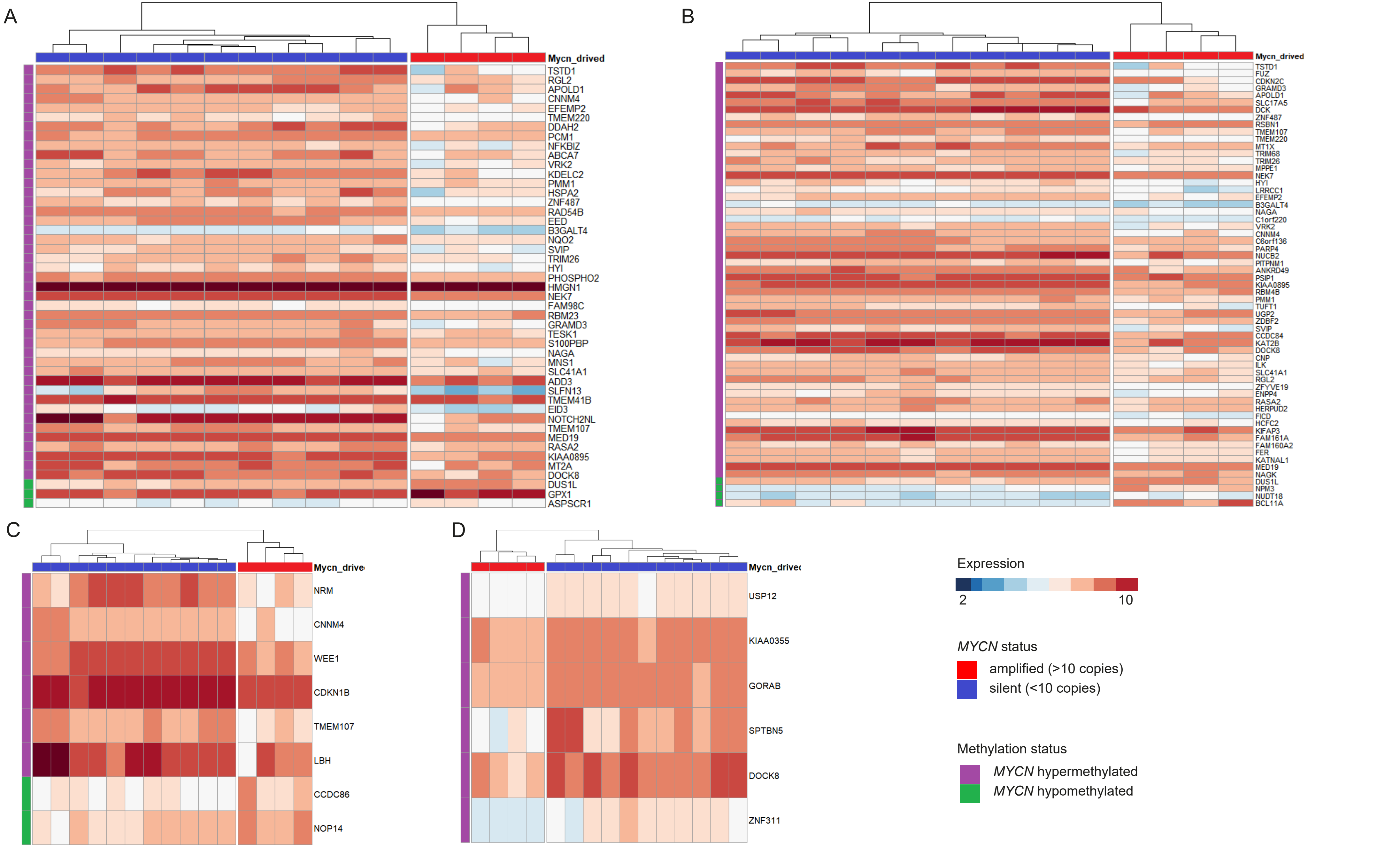

Supplementary figure 4. Methylation driven differential expression in *MYCN*-amplified tumours (N=4) vs. *MYCN* silent (N=11). Hierarchical clustering with heat maps depicts the results from the integrated analysis primarily performed for 15 retinoblastoma samples with matched expression and methylation data. Set of genes which are differentially- methylated, differentially-expressed and show significant gene expression-methylation correlation in *MYCN*-amplified retinoblastomas compared to *MYCN*-silent retinoblastomas. Four independent analyses were carried out for four methylation sites in relation to CpG islands **A)** Promoter island **B)** promoter shore **C)** promoter shelf **D)** promoter Open sea

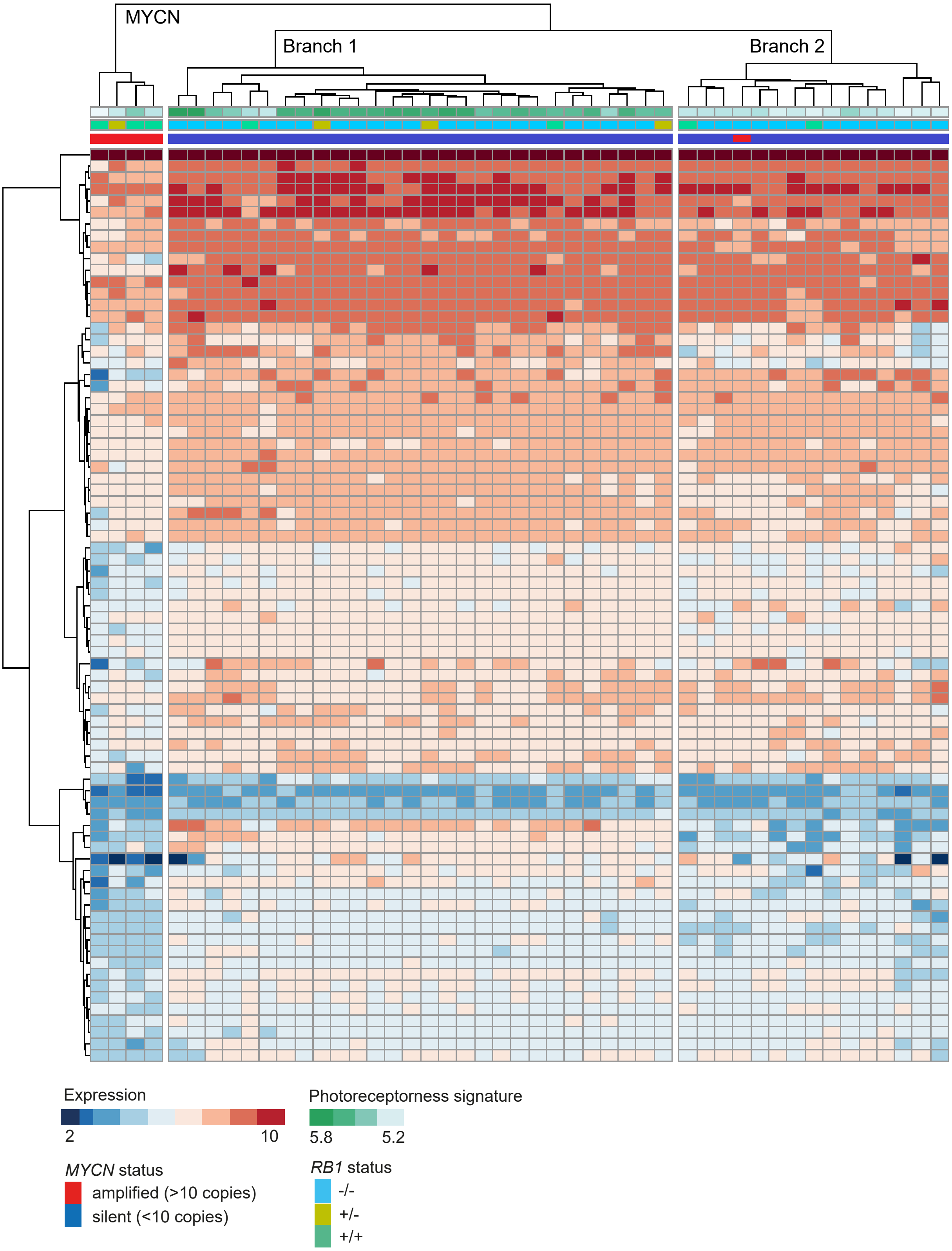

Supplementary figure 5. Hierarchical clustering with heatmap for 79 hypermethylation downregulated genes in *MYCN*-amplified *RB1*-proficient samples

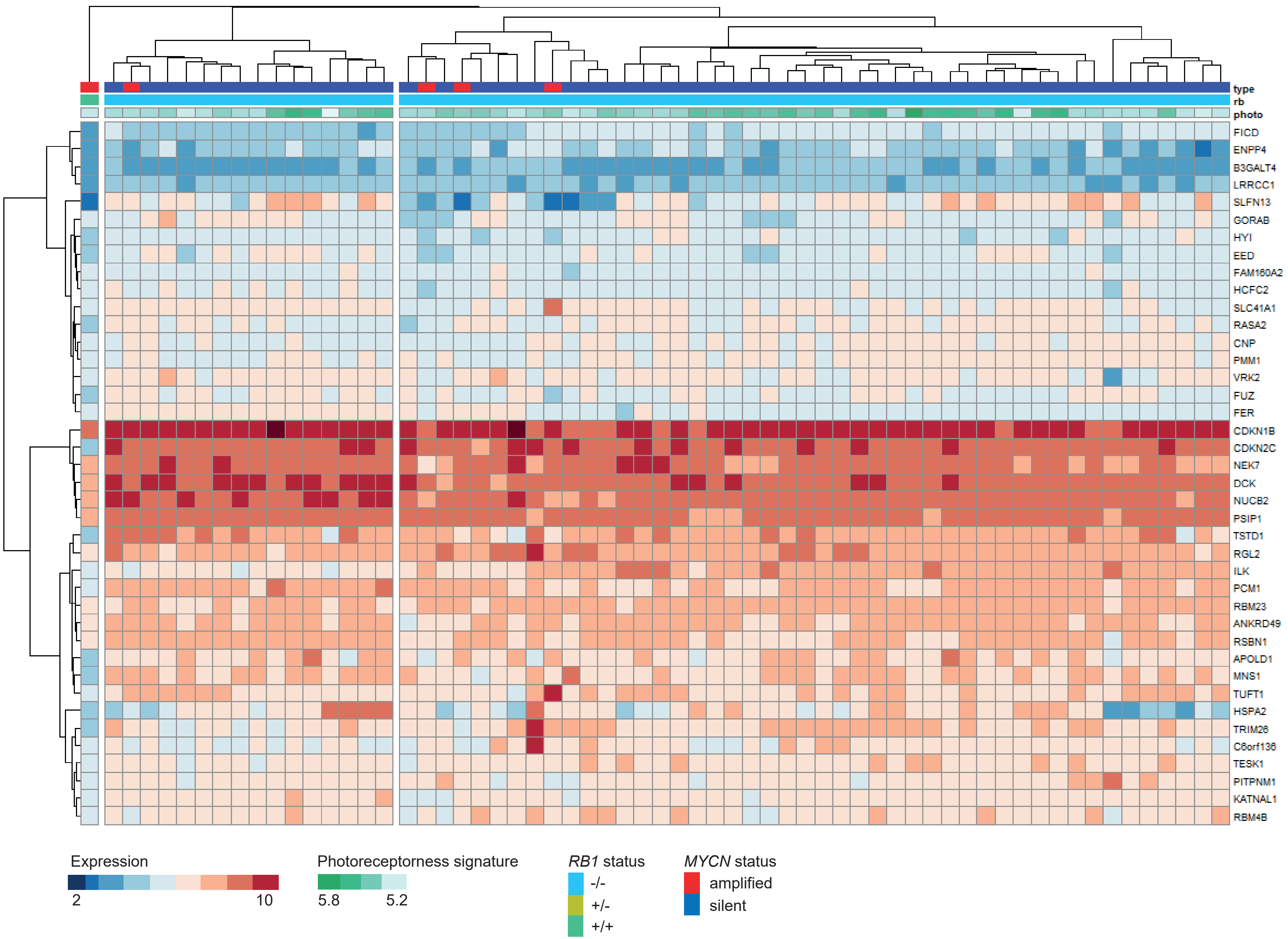

Supplementary figure 6. The expression of 40 Hyper-methylation driven genes in an independent publicly available retinoblastoma cohort (N=62). Hierarchal clustering (WARD2) with heatmap showing the relationship between samples for the expression of 40 genes. in agreement with the findings in the current study, the *MYCN*-amplified *RB1*--proficient retinoblastoma exhibits a distinct expression pattern for these genes and deviates from *RB1* deficient tumours with or without *MYCN* amplification.
